# Enhancing organic and inorganic carbon sequestration in calcareous soil by the combination of wheat straw and wood ash and/or lime

**DOI:** 10.1371/journal.pone.0205361

**Published:** 2018-10-10

**Authors:** Huili Zhao, Huijie Zhang, Abdul Ghaffar Shar, Jifei Liu, Yanlong Chen, Songjie Chu, Xiaohong Tian

**Affiliations:** 1 College of Natural Resources and Environment/Key Lab of Plant Nutrition and the Agri-environment in Northwest China, Ministry of Agriculture, Northwest A&F University, Yangling, Shaanxi, China; 2 Heyang Field Station of Agricultural Environment and Farmland Conservation, Ministry of Agriculture, Fuping, Shaanxi, China; Beijing Normal University, CHINA

## Abstract

Increasing organic carbon sequestration in agricultural soils is important for improving soil fertility and mitigating climate change. Wood ash is generally applied as a potassium fertilizer, but the effects of simultaneous incorporation of wood ash and crop straw on the turnover of soil organic carbon (SOC) and inorganic carbon (SIC) are not well understood. In this study, a 118-day lab incubation experiment was conducted using a calcareous soil (with 10 years of continuous maize cropping history) to study the effects of adding wheat straw, wood ash and lime. Our study showed that straw addition led to an increase in both SOC (19%) and SIC (3%). Wood ash and lime addition decreased CO_2_ emission by 182 and 1210 mg kg^-1^ and increased SIC by 125 and 1001 mg kg^-1^ during the incubation, respectively, which was due to supply of CaO from wood ash and lime. The increase of SOC content was 2.4% due to the addition of lime. In addition to straw addition enhanced straw-derived OC content, the addition of lime also increased straw-derived OC content by 34.5%. This study demonstrated that lime was more effective in reducing CO_2_ emission and and enhancing SOC than wood ash. In conclusion, adding lime to calcareous soil might be an effective method of enhancing carbon sequestration and slowing climate change.

## Introduction

Agricultural soils can mitigate the emission of greenhouse gases and enhance soil sustainability, so the potential for carbon sequestration in agricultural soils has received significant attention [1]. Soil organic carbon (SOC) and inorganic carbon (SIC) are important carbon reservoirs in arid and semi-arid regions that play an important role in the global carbon cycle and climate change [2]. However, soil organic carbon has attracted much attention, and SIC has received much less attention despite its potential for carbon sequestration. Wang et al. [3] reported that the SOC and SIC stocks were greater in agricultural land than in non-agricultural land in arid and semi-arid regions. Many reports showed that long-term straw incorporation and manure application in arid cropland led to SOC enhancement and carbon sequestration in the form of carbonate [4–6]. These results suggest that there are many ways to increase SIC content in agricultural soils.

SOC content in soils can be enhanced by commonly used best management practices, including the utilization of minimum or no tillage, cover crops, organic amendments and balance fertilization [7, 8]. Soil organic carbon sequestration, which relies on traditional management practices, is not always effective because of the loss of applied organic C and native soil C [8]. For example, Zhao et al. [9] reported that the cumulative C input achieved by straw return was much higher than that observed without straw return; however, the corresponding increases in SOC stock under straw return were relatively minor, which indicated a substantial loss of C input. Therefore, research is required to determine how CO_2_ released from straw can be fixed to form inorganic carbon in the context of widespread application of straw returning measures. In addition, Kirkby et al. [10] showed that the amount of straw converted into “new” fine fraction soil organic matter (SOM) was increased by up to three-fold by augmenting the residues with supplementary nutrients. Thus, the development of methods of fixing more carbon from straw into soil has significant potential to improve soil fertility and reduce CO_2_ emissions. Jastrow et al. [11] reported that the application of soil amendments with a high specific area enhanced SOC content via physico-chemical protection of SOC by organo-mineral complexation. Previous studies suggest that fly ash increases SOC content because of its large specific area [11]. In addition, the presence of CaO, Ca(OH) and MgO in fly ash results in uptake of CO_2_ by the soil as a consequence of carbonation reactions [12]. These studies suggest that the application of mineral amendments, such as wood ash, with its high specific surface area and metal oxide content, with organic residue could enhance soil C content [13]. In addition, revealing soil C dynamics under long-term straw return is essential to understanding changes in new soil C inputs and mineralization of old C. SOC sources can be determined because the δ^13^C values of C_3_ (δ^13^C *ca*.−28‰) and C_4_ (δ^13^C *ca*.−12‰) vegetation are different because of differences in C isotope utilization [14]. The relative contribution of new SOC and old SOC can be estimated based on the mass of each C isotope [14]. In the present study, new soil C formation was quantified by measuring δ^13^C abundance based on changes in decomposition after 10 years of continuous corn planting.

The large quantities of wood ash generated by the wood industry and the increasing cost of wood ash storage have encouraged the search for alternative uses [15]. Recycling biomass ash in agriculture may solve the problem of disposal and reduce the doses of commercial fertilizer required for crops [16]. Wood ash is characterized by its high alkaline metal content and generally contains significant quantities of Ca, Mg and K [17]. Reductions in soil acidity and increased base saturation have been widely reported after the application of wood ash to forest mineral soils [18, 19]. Traditionally, the major use of wood ash in China has been as a potassium fertilizer [20, 21]. Although calcareous soils are common in arid and semi-arid climates and constitute more than 730×10^6^ ha of soil worldwide, information on the value of ash fertilizers in calcareous soils is scarce, especially when they are used in combination with straw [22]. In calcareous soils, carbonization of Ca and Mg present in wood ash fixes CO_2_ produced from soil respiration and thus reduces CO_2_ emissions while increasing SOC content [23]. However, Maljanen et al. [24] reported that wood ash resulted in greater CO_2_ fluxes in acidified forest soils by stimulating the microbial population. These conflicting results may be due to differences in initial soil pH, ash application rates, ash types, soil types, and inputs of organic matter that occurred in these various studies. In addition, Kleber et al. [25] showed that the minerals and metals in wood ash can protect SOM from decomposition by interacting with it. Conversely, Hansen et al. [26] reported that the liming effect and the input of nutrients in response to wood ash could provide more favorable conditions for soil microbes, which could expedite decay of SOM in acidic soil. However, the effect of the combination of wood ash and crop straw on SOC content has not been evaluated comprehensively in alkaline soil, and little is known regarding the effect of wood ash on straw-derived C sequestered in alkaline soil. Traditionally, lime materials, such as calcite (CaCO_3_), burnt lime (CaO), and dolomite [CaMg(CO_3_)_2_] are used to neutralize acidic soils [27, 28]. In alkaline calcareous soils, CaCO_3_ is added as a net sink of CO_2_, whereas CaCO_3_ functions as a net source of CO_2_ in acidic soils [29]. Certainly the presence of CaO, Ca(OH), and MgO will enhance uptake of CO_2_ by the soil as a consequence of carbonation reactions [12]. A previous study conducted under controlled conditions (laboratory incubation experiments) suggested that wood ash reduced CO_2_ emission [23] due to the formation of inorganic carbon. We believe that the presence of large amounts of oxides in wood ash will lead to carbonation of Ca and Mg as follows: (Ca^2+^ or Mg^2+^) + CO_3_^2−^ → CaCO_3_ or MgCO_3_. For the sake of verifying the function of wood ash materials for CO_2_ capture, we selected the higher content of CaO in wood ash as a reference to test the mechanism, so we set up another lime treatment; moreover, the added lime amount was the same as the amount of CaO in the wood ash. The addition of lime to soil could increase the solubility of dissolved organic carbon (DOC) and enhance DOC leaching because of deprotonation or desorption [30]. Ahmad et al. [31] reported that liming-induced decreases in SOC were mainly attributed to enhanced C mineralization following an increase in C solubility. Therefore, liming could increase or decrease the DOC concentration in soil depending on which processes dominate. Andersson et al. [30] also demonstrated that the DOC concentration is more dependent on soil pH than on soil microbial activity, and a rapid increase in soil pH may increase the solubility of organic matter.

We hypothesized that adding wood ash and/or lime in the presence of straw would reduce CO_2_ emission, enhance SIC formation through CaO hydration, directly increase SOC stocks by increasing the abundance of Ca^2+^ ions (which function as soil aggregation agents), and indirectly affect SOC by changing soil properties. The combination of wood ash and lime significantly increased the amount of straw-derived C, so, we performed laboratory incubation experiments using calcareous soil to assess the effects of straw, wood ash and lime on CO_2_ mineralization, SOC content, SIC content and soil chemical properties.

## Materials and methods

### Characterization of soil, wheat straw, wood ash and lime

We confirm that the owner of the land, Northwest A&F University, gave permission to conduct the study on this site. Our field studies did not involve endangered or protected species. The soil used in this experiment was taken from the topsoil (0–20 cm) of an agricultural field at the Changwu Agricultural and Ecological Experimental Station (35.14° N, 107.40° E; 1152 m a.s.l.) on the Loess Plateau in northwestern China. The soil samples were air dried and passed through a 2-mm sieve to remove rocks, coarse crop residues and roots. The soil samples were obtained from a monocrop planting area in which maize (C_4_ crop) was grown for ten years. The soil at the site was a silt clay loam (according to the Chinese Soil Taxonomy) that contained 5.4% sand, 51.7% silt and 42.9% clay; the soil at the site was tentatively classified as Earth-cumuli-Orthic Anthrosol [32]. The soil samples had the following soil properties: pH 8.4, SOC 8.91 g kg^-1^, total nitrogen 1.2 g kg^-1^, microbial biomass C 163 mg kg^-1^, DOC 32.4 mg kg^-1^, available phosphorus 18.4 mg kg^-1^, available potassium 152 mg kg^-1^, δ^13^C -19.5‰, CaCO_3_ 67 g kg^-1^.

The wheat stem (internode) material used in this study was collected at harvest at the Doukou Experimental Station of Northwest A&F University, oven-dried at 70°C, and stored. The wheat straw had an average C content of 456 g kg^-1^ and a mean total N content of 6.8 g kg^-1^, which had a δ^13^C value of -27.5‰. The straw was cut into pieces approximately 2-cm long prior to mixing with the soil.

Dust-like wood ash was produced from the branch of a kiwi fruit tree (*Actinidia*) by combustion in a home fireplace. The wood ash was dried at 50°C and sieved through a 0.25-mm mesh screen prior to use and analysis. The wood ash had a δ^13^C value of -26.4‰. The chemical properties of the wood ash are given in Table 1.

**Table 1:** Chemical properties of wood ash used in the experiment. All data are expressed on a dry weight basis.

Property	Value
pH	12.44
EC (dS/m)	24.75
Organic carbon (g kg^-1^)	3.2
Total carbon^§^ (g kg^-1^)	39
Total nitrogen (g kg^-1^)	0.75
SOC:TN^∮^	4.3
CaO (g kg^-1^)	434.0
K_2_O (g kg^-1^)	150.9
SiO_2_ (g kg^-1^)	123.4
MgO (g kg^-1^)	98.4
P_2_O_5_ (g kg^-1^)	58.7
Al_2_O_3_ (g kg^-1^)	44.9
Fe_2_O_3_ (g kg^-1^)	24.5
Na_2_O (g kg^-1^)	10.4
Cl (g kg^-1^)	7.1

^§^represents soil organic carbon plus soil inorganic carbon

^∮^represents the ratio of soil organic carbon to total nitrogen

The lime used in the experiment contained an amount of CaO equivalent to that contained in the wood ash.

### Experimental design

An incubation experiment was conducted using a completely randomized design. A 2 × 2 × 2 factorial experiment corresponding to two levels of wood ash addition (no wood ash, W_0_; 12 g wood ash kg^-1^ soil, W_12_), two levels of lime addition (no lime, L_0_; 5 g lime kg^-1^ soil, L_5_) and two levels of straw addition (no straw, S_0_; 15 g straw kg^-1^ soil, S_15_) was established. Each treatment had three replicates.

Fresh soil samples (250 g dried weight) were placed in 1-L plastic jars and pre-incubated for 7 days. The wood, lime and/or straw were mixed thoroughly with the soil samples in each jar before a solution containing N and P was added. Urea and diammonium phosphate were dissolved in deionized water and added in the form of a solution (4.4 g L^-1^ N, 2.1 g L^-1^ P_2_O_5_, 5 mL to each glass jar), and the soil moisture content was adjusted to 70% of the soil water holding capacity using deionized water. During the 118-day incubation period, the temperature was maintained at 25 ± 1°C. The weight of each sample was recorded at the beginning of the treatment, and the water lost by evaporation was replaced with deionized water every five days. Three replicates per treatment group were destructively sampled to determine the soil DOC and pH at days 2, 10, 45, and 118. In order to allow destructive measurements of different experimental units on each sampling date [33], we prepared 96 experimental units (8 treatments × 4 sampling dates × 3 replicates).

### Measurement of soil CO_2_ effluxes and analysis

An open plastic vial containing 20 mL of 1 M sodium hydroxide (NaOH) was placed in the jar containing soil to absorb CO_2_ respired during the subsequent incubation. Four blank jars containing only water and NaOH were also set up as above. Soil CO_2_ efflux was assessed on days 2, 3, 4, 5, 7, 10, 15, 20, 25, 35, 45, 65, 95, and 118. The NaOH vials were replaced with fresh vials on each test day. The jars were opened at each measurement of CO_2_ emission to allow air exchange. The trapped CO_2_ was determined as total CO_2_ evolved by titrating alkali to a phenolphthalein end point with 0.5 M HCl [34] and expressed as mg C kg^−1^ soil.

At the completion of the incubation period, all of the samples were ground through a 2-mm sieve. After mixing thoroughly, one subsample of the sieved soil was air-dried for soil analysis, while another subsample was stored at 4°C for DOC analysis. The DOC was determined by the method of Jones and Willett [35]. C concentrations in the extracts were measured using a Multi 2011 N/C TOC analyzer (Analytik Jena, Germany). The soil pH was determined using a glass electrode meter in soil/water suspensions (1:2.5 soil:water). At the completion of the 118-day incubation period, any C remaining in the samples was again assumed to be SOC (determined by the wet oxidation method) after any partially degraded wheat straw remaining in the jars was removed by the dry-sieving winnowing method [36]. In order to measure the amount of C in the remaining straw, three replicates of each treatment were prepared in another 12 jars (4 treatments and 3 replicates). Briefly, each mixture of soil and straw was poured into water and then sufficiently stirred so that the straw floated in the water, after which the undecomposed straw was filtered using a sieve, and the process was repeated again with fresh water. Finally, the partially degraded straw was dried and weighed. The C content of the remaining straw was calculated as the residual straw weight multiplied by the residual straw carbon content.

Soil inorganic carbon was measured as described by Bao et al. [37]. The δ^13^C values of wheat straw, wood ash and SOC were determined using a dry combustion analyzer with an attached isotope ratio mass spectrometer (Europa Scientific Model 20–20). 1 g soil was pretreated with 10 mL 1 M HCl for 12 hours to remove carbonate. The precision for δ^13^C was ± 0.10‰ based on repeated measurements of a working standard. The δ^13^C of the samples was expressed as following:

$$\delta^{13}{C(‰)=[(R}_{\mathrm{sample}}{/R}_{\mathrm{PDP}})‑1]\times 1000$$ (1)

where *R*_sample_ is the ^13^C/^12^C ratio of the sample and *R*_PDB_ is the ^13^C/^12^C ratio of the Pee Dee Belemnite (PDB) standard [38].

### Data calculation

The ratio of straw-derived C to SOC (f_new_) from the incubated jars over 118 days was calculated using a two-component isotopic mixing model [39].

$$f_{\mathrm{new}}{(\%)=(\delta }^{13}C_{\mathrm{soc}-a}-\delta^{13}C_{\mathrm{soc}-b})/(\delta^{13}C_{\mathrm{material}}-\delta^{13}C_{\mathrm{soc}-b})$$ (2)

where δ^13^C_soc-a_ is the δ^13^C of SOC in straw/lime/wood ash-amended soils after the incubation, δ^13^C_soc-b_ is the δ^13^C of SOC in non-amended soil before the incubation, and δ^13^C_material_ is the δ^13^C of the straw/wood ash mixture.

$$\mathrm{New}\;C=f_{\mathrm{new}}(\%)\times \mathrm{Total}\;\mathrm{SOC}$$ (3)

NativeC=TotalSOC−NewC (4)

### Statistical analysis

All reported values are the means of 3 replicate jars. Data Processing System (DPS) version 7.05 statistical software (Ruifeng Information Technology Co., Ltd., Hangzhou, China) was used for the statistical analysis. The significance of the effects of the straw, wood ash and lime treatments, as well as their interactions on the reported traits, were evaluated by analysis of variance (ANOVA). All statistical differences were calculated by ANOVA, and the means were segregated by the LSD multiple comparison test at P < 0.05.

## Results

### Soil CO_2_ emission

**Fig 1:**
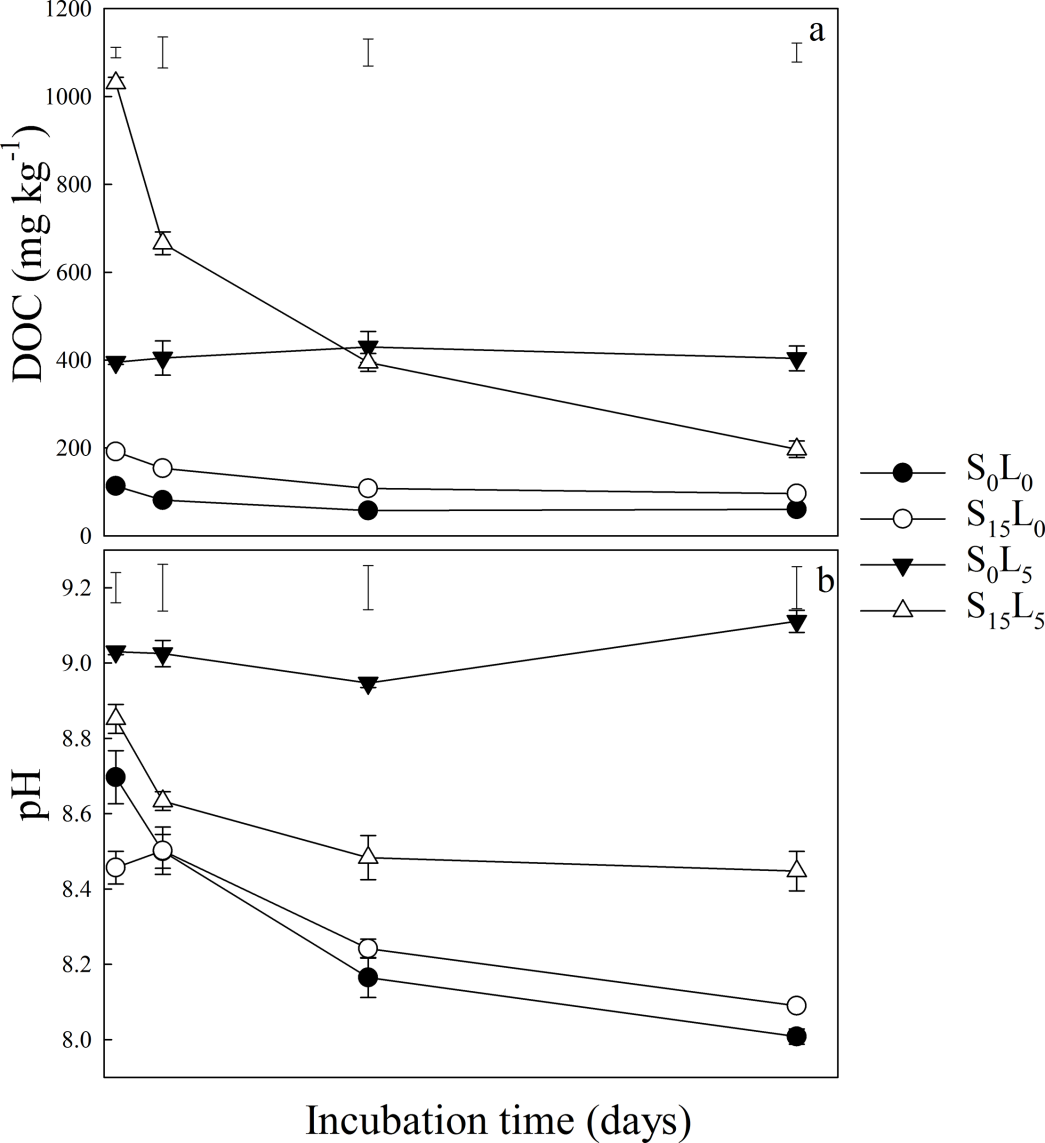
**CO**_**2**_**-C emission rate (a) and cumulative CO**_**2**_**-C emission (b) during straw decomposition under different treatments.** (S_0_W_0_L_0_, only soil; S_0_W_12_L_0_, only wood ash; S_0_W_0_L_5_, only lime; S_0_W_12_L_5_, wood ash plus lime; S_15_W_0_L_0_, only wheat straw; S_15_W_12_L_0_, wheat straw plus wood ash; S_15_W_0_L_5_, wheat straw plus lime; S_15_W_12_L_5_, wheat straw and wood ash plus lime.). The error bars outside the curve represent LSD.

Unsurprisingly, in this study, the CO_2_ emission rate from wheat straw-amended soil was much higher than that of soil with no straw added during the entire incubation (Fig 1A). Cumulative CO2 emission was significantly affected by main effects of wood ash and lime addition (Table 2). With no wood ash or lime addition (S_0_W_0_L_0_, S_15_W_0_L_0_), the soil CO_2_ emission rates peaked on the second day of the incubation, and decreased with time. Incorporation of wood ash or lime into the soil postponed the peak occurrence of the CO_2_ emission rate whether or not straw was added (Fig 1A). Incorporation of wood ash or lime into the soil decreased the CO_2_ emission rate. However, in soils with no straw addition, adding only wood ash decreased the CO_2_ emission rate mainly during the first 5 days of the incubation relative to that of the soil with no amendment, whereas adding lime reduced the CO_2_ emission rate during the entire incubation period (118 days). In the soil with straw amendment, adding only wood ash reduced the CO_2_ emission rate only on the first day of the incubation, whereas the reduction in the CO_2_ emission rate produced by adding lime persisted for the first ten days (Fig 1A). Soil amended with straw had much greater cumulative CO_2_ emission than that of soil with no addition (p < 0.05, Fig 1B). Whether or not straw was added, the addition of wood ash and/or lime decreased cumulative CO_2_ emission compared with that of soil with no amendment. The cumulative reduction in CO_2_ emission from soil with lime was 5.4 times that of soil with wood ash (p < 0.05, Table 3).

**Table 2:** Cumulative CO_2_ emission, final SOC, final SIC, soil DOC and pH at each sampling time.

Time	Variation	CO_2_	Final	Final		
(days)	source	emission^§^	SOC	SIC	DOC^a^	pH
10	Straw (a)	<0.001	-	-	<0.001	<0.001
	Wood ash (b)	NS	-	-	NS	NS
	Lime (c)	<0.001	-	-	<0.001	<0.001
	a × b	NS	-	-	NS	NS
	b × c	<0.001	-	-	NS	NS
	a × c	<0.001	-	-	0.029	<0.001
	a × b × c	NS	-	-	NS	NS
45	Straw (a)	<0.001	-	-	NS	<0.001
	Wood ash (b)	0.008	-	-	NS	<0.001
	Lime (c)	<0.001	-	-	<0.001	<0.001
	a × b	NS	-	-	NS	NS
	b × c	<0.001	-	-	NS	NS
	a × c	<0.001	-	-	NS	<0.001
	a × b × c	NS	-	-	NS	NS
118	Straw (a)	<0.001	<0.001	0.004	<0.001	<0.001
	Wood ash (b)	<0.001	NS	0.008	0.002	<0.001
	Lime (c)	<0.001	0.004	<0.001	<0.001	<0.001
	a × b	NS	0.002	NS	0.031	NS
	b × c	0.004	NS	NS	NS	0.044
	a × c	<0.001	NS	0.005	<0.001	<0.001
	a × b × c	NS	NS	NS	0.019	NS

NS, not significant

^§^represents cumulative CO_2_ emission at each sampling time.

^a^ DOC, dissolved organic carbon

**Table 3:** Effects of straw, wood ash and lime amendment on cumulative CO_2_ emission, final SOC, final SIC and δ^13^C after the incubation.

Treatment	CO_2_ emission	Increase amount	Increase rate (%)	Final net SOC	Increase amount	Increase rate (%)	Final SIC	Increase amount	Increase rate (%)	δ^13^C (%)	Increase rate (%)
	(mg kg^-1^)			(mg kg^-1^)			(mg kg^-1^)				
Straw											
S_0_	428b	-	-	8377b	-	-	8852b	-	-	-20.5a	-
S_15_	3978a	3550	829%	9974a	1597	19%	9119a	267	3.0%	-21.6b	-5.4%
Wood ash (W)											
W_0_	2294a	-	-	9161a	-	-	8923b	-	-	-21.0a	-
W_12_	2112b	-182	-7.9%	9189a	28	0.3%	9048a	125	1.4%	-21.1a	0.5%
Lime (L)											
L_0_	2808a	-	-	9066b	-	-	8485b	-	-	-20.9a	-
L_5_	1598b	-1210	-43%	9284a	218	2.4%	9486a	1001	11.8%	-21.3b	-1.9%

Mean values having different lower-case letters are significantly different among straw rate, wood ash rate or lime rate (P ≤ 0.05).

### SOC, straw-derived new OC, and SIC

In soil with no straw amendment, the net SOC content at the end of the incubation was reduced compared to the initial value because of mineralization (9317 mg C kg^-1^ soil, Table 4). The reductions in net SOC content produced by the S_0_W_0_L_0_, S_0_W_0_L_5_, S_0_W_12_L_0_, and S_0_W_12_L_5_ treatments were 9.6%, 9.8%, 10.4%, and 10.5%, respectively; In soil with straw amendment, the net SOC content was increased compared to the initial SOC (by 3.7%, 9.1%, 5.6%, and 9.9% by S_15_W_0_L_0_, S_15_W_0_L_5_, S_15_W_12_L_0_, and S_15_W_12_L_5_, respectively). On average, straw addition increased net SOC content by 19% compared to that of soil with no amendment. In addition, lime addition increased the average net SOC content by 2.4% compared to that of soil with no amendment (p < 0.05, Table 3). At the end of the incubation, in the case of straw addition, the organic carbon of residual straw mixed in the soil was reduced by 47.4% in soil with lime amendment (S_15_W_0_L_5_, S_15_W_12_L_5_) relative to that of soil with no lime amendment (S_15_W_0_L_0_, S_15_W_12_L_0_) (p<0.05, Table 4).

**Table 4:** Change in organic C content (mg C kg^-1^ soil) caused by the treatments before and after the incubation.

Treatment					Input		Lost		
			Final^‡^	SOC increase relative to control	Organic C in straw	Organic C in wood ash	Cumulative CO_2_-C emission	CO_2_ emission reduction relative to control	Organic C in remaining straw
S_0_	W_0_	L_0_	8422c	-^†^	-	-	927d	-^※^	-
		L_5_	8399c	-23c	-	-	85f	-842b	-
	W_12_	L_0_	8350c	-72c	-	38	680e	-247a	-
		L_5_	8337c	-85c	-	38	20f	-907b	-
S_15_	W_0_	L_0_	9660b	-^†^	6845	-	4991a	-^※^	1378a
		L_5_	10166a	506a	6845	-	3173c	-1818b	677b
	W_12_	L_0_	9836b	176b	6845	38	4637b	-354a	1442a
		L_5_	10237a	577a	6845	38	3111c	-1880b	805b

Initial SOC content is 9317 mg C kg^-1^ soil.

^‡^Soils with any partially degraded straw removed.

^†^Represents SOC formation in soil amended with exogenous substances relative to that of the S_0_W_0_L_0_ and S_15_W_0_L_0_ soils.

^※^Represents reduced CO_2_ emission relative to that in the S_0_W_0_L_0_ and S_15_W_0_L_0_ soils.

Different lower-case letters indicate significant differences among the treatments (P < 0.05).

The addition of straw reduced the δ^13^C of SOC value by 5.4% relative to that of soil with no amendment. Moreover, adding lime reduced the δ^13^C of SOC value by 1.9% relative to that of soil with no amendment (p < 0.05, Table 3). Accordingly, the average new straw-derived OC was 2832 mg C kg^-1^ soil. In particular, adding lime increased new OC by 34.6% compared to that of soil with no amendment (p < 0.05, Table 5).

**Table 5:** Effects of straw, wood ash and lime amendment rate on the δ^13^C signature of soil organic carbon ratio of straw-derived OC to SOC (f_new_, %), ratio of native soil-derived OC to SOC (f_native_, %), straw-derived OC amount (New OC) and native soil-derived OC (Native SOC) with wheat straw addition.

Treatment			δ^13^C	f_new_	New OC	f_native_	Native SOC
			(‰)	(%)	(mg·kg^-1^)	(%)	(mg·kg^-1^)
S_0_	W_0_	L_0_	-20.38	0	0	100	8423
		L_5_	-20.70	0	0	100	8151
	W_12_	L_0_	-20.35	0	0	100	8355
		L_5_	-20.58	0	0	100	8217
S_15_	W_0_	L_0_	-21.37a	23.9b	2310c	76.1	7351a
		L_5_	-21.64b	27.3b	2780b	72.7	7392a
	W_12_	L_0_	-21.36a	25.6b	2520bc	74.4	7321a
		L_5_	-22.18c	36.4a	3720a	63.6	6489b

Mean values with different lower-case letters are significantly different among the straw addition treatments (P < 0.05).

The final SIC content was affected by adding straw, wood ash or lime (p < 0.05, Table 2). Lime increased SIC by 11.8% in comparison with that of soil without lime, whereas wood ash increased SIC by 1.4% in comparison with that of soil without wood ash (p < 0.05, Table 3). In soil with no straw amendment, the net SIC content at the end of the incubation was increased compared to its initial value. In soil with straw amendment, the net SIC content at the end of the incubation was further increased compared to its initial value, with the exception of soil with straw only (p < 0.05, Table 6).

**Table 6:** Change in inorganic C content (mg C kg^-1^ soil) caused by the treatments before and after the incubation.

Treatment			SIC Final	Inorganic carbon in wood ash	Net △SIC^∮^	SIC increase relative to control
S_0_	W_0_	L_0_	8360d	-	-30d	-^†^
		L_5_	9212c	-	822b	852a
	W_12_	L_0_	9039c	432	217c	247b
		L_5_	9660b	432	838b	868a
S_15_	W_0_	L_0_	8377d	-	-13d	-^†^
		L_5_	9743b	-	1353a	1353a
	W_12_	L_0_	9029c	432	207c	220b
		L_5_	10192a	432	1370a	1383a

Initial SIC content is 8390 mg C kg^-1^ soil.

^∮^Represents the final SIC after the incubation minus the initial SIC content and inorganic carbon from wood ash itself.

^†^Represents SIC formation in soil amended with an exogenous substance relative to that in the S_0_W_0_L_0_ and S_15_W_0_L_0_ soils. Different lower-case letters indicate significant differences among the treatments (P < 0.05).

### Soil pH and DOC

**Fig 2:**
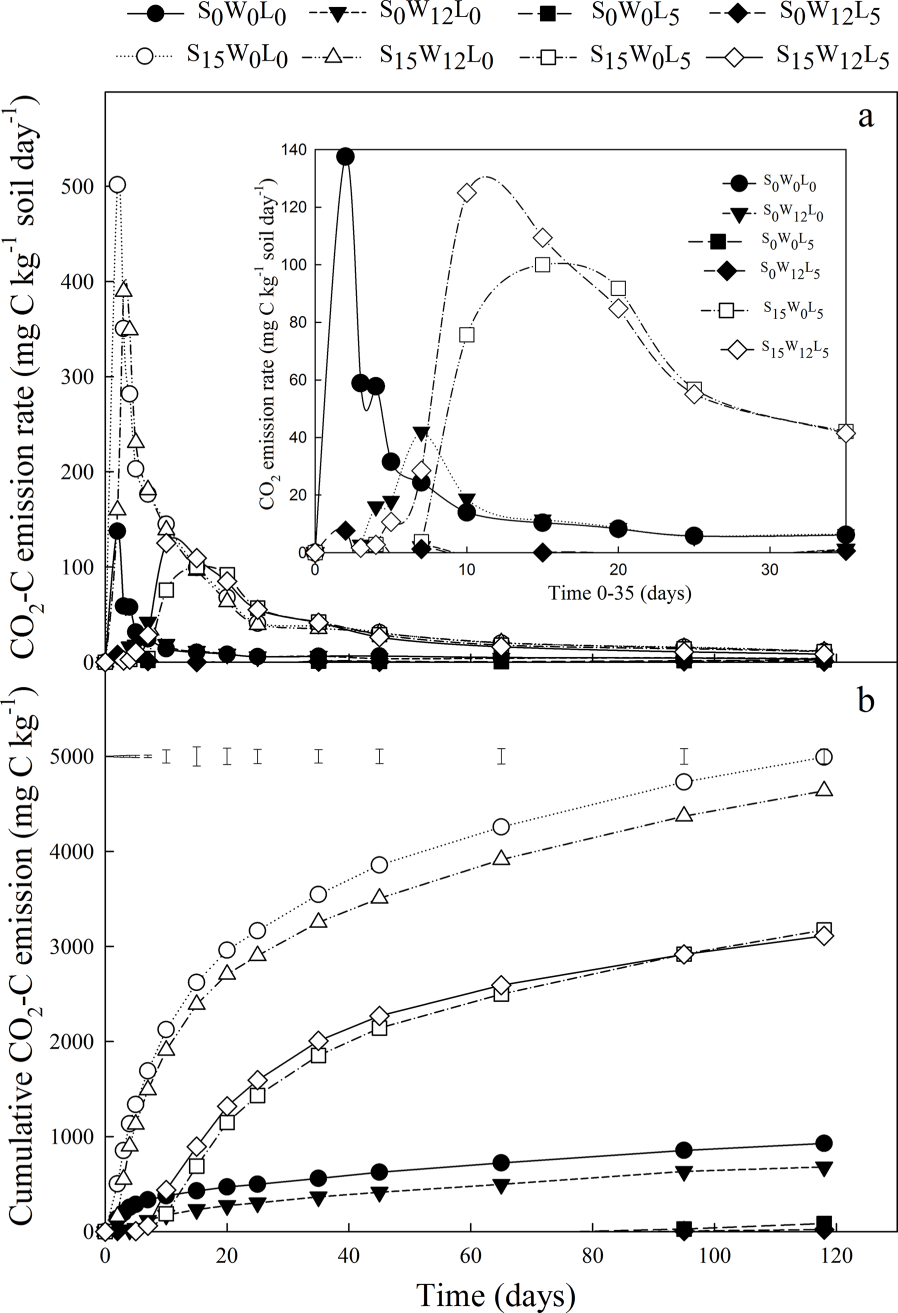
Effects of the amendment rates of straw and lime on dissolved organic carbon (DOC) and soil pH. S_0_L_0_, including S_0_W_0_L_0_ and S_0_W_12_L_0_; S_15_L_0_, including S_15_W_0_L_0_ and S_15_W_12_L_0_; S_0_W_0_L_5_, including S_0_W_0_L_5_ and S_0_W_12_L_5_; S_15_W_0_L_5_, including S_15_W_0_L_5_ and S_15_W_12_L_5_. The error bars outside the curve represent LSD.

Irrespective of whether straw was added, the addition of lime greatly increased the DOC content in comparison with that of soil with no amendment after different periods of incubation (p < 0.05, Fig 2). In contrast, the addition of wood ash significantly increased DOC content on day 118 by 21.6% compared to that of soil with no addition, whereas the soil DOC content was unchanged on the other test days (p < 0.05, Table 7).

**Table 7:** Effect of the wood ash amendment rate on soil DOC and pH after 2, 10, 45 and 118 days of incubation.

Incubation time (days)	Treatment	DOC (mg kg^-1^)	Soil pH
2	W_0_	432a	8.65b
	W_12_	433a	8.86a
10	W_0_	317a	8.62a
	W_12_	336a	8.70a
45	W_0_	262a	8.35b
	W_12_	233a	8.56a
118	W_0_	171b	8.32b
	W_12_	208a	8.50a

Mean values with different lower-case letters are significantly different (P < 0.05).

During the entire incubation, straw decreased soil pH, but lime increased soil pH. In contrast, wood ash increased soil pH only during the late period of incubation (p < 0.05, Table 7). When no straw was added, lime amendment caused the largest increase in soil pH among the tested treatments (0.68 on average in comparison with soil without lime), whereas the addition of straw increased the soil pH by only 0.28 in comparison with that of soil without straw (Fig 2B).

## Discussion

### Potential mechanism of the reduction in CO_2_ emission caused by wood ash and lime

According to a previous study conducted in our laboratory, the simultaneous addition of straw and wood ash decreased cumulative CO_2_ emission by approximately 6.2% compared to the addition of straw only. In the present study, the extent of the reduction of cumulative CO_2_ emission due to wood ash amendment (7.9%, Table 3) was very similar to that from our previous study, but the addition of wood ash had no obvious effect on SOC sequestration. We surmised that the high content of CaO in wood ash (434 g kg^-1^) might be the main reason for the lack of an observable effect of wood ash amendment on SOC sequestration [23]. Therefore, in the present study, experiments were conducted using lime (CaO) to determine whether CaCO_3_ formed from CaO and CO_2_ emitted during the incubation was responsible for the reduction in CO_2_ emission. Moreover, the rate of CaO added to the soil using lime was equivalent to that added by wood ash amendment.

In the present study, lime amendment led to much less accumulative CO_2_ emission than did wood ash (Fig 1; Table 3). When no straw was added to the soil, the period of CO_2_ emission reduction caused by wood ash (5 d) was much shorter than that caused by lime (118 d). When straw was added to the soil, the periods of CO_2_ emission reduction caused by wood ash (1 d) and lime (10 d) were both shortened. Several factors could be responsible for this phenomenon. First, when CO_2_ emission was low (no straw added), the CaO contained in wood ash and lime was depleted for a relatively long period; however, when CO_2_ emission was high (after straw amendment), CaO from lime and wood ash was exhausted more rapidly. Second, although the CaO content of wood ash was as high as 43%, and the rate of CaO addition from lime was kept at 43% to attempt to match the amount of CaO added by the wood ash amendment, the actual amount of CaO in wood ash may have been much lower than this value. This difference in the assumed and actual CaO content of wood ash may have been the primary reason why the reduction in CO_2_ emission caused by wood ash was much shorter than that caused by lime. Another finding supporting this notion is the measurement of inorganic carbon, present in the form of CaCO_3_; the increase in net SIC following the addition of wood ash was much smaller than that observed following the addition of lime (Table 3). Future studies should determine the true proportions of CaO and other forms of Ca in wood ash. The findings described above were all obtained from experiments using calcareous soils. However, Pugliese et al. [40] and Ohlsson [41] found similar results using acidic forest soil, whereas Zimmermann et al. [42] showed that wood ash amendment in acidic forest soil resulted in significant increases in the rate of CO_2_ evolution and microbial biomass C due to an increase in microbial activity, which was related to increases in the pH value and quantity of nutrients over the 460-day experiment. These differences in the findings from different studies are likely due to differences in initial soil pH, ash types, ash application rate, and other experimental parameters.

This study showed that lime and wood ash amendment significantly increased SIC content (Table 3). The major reason for the reduction in CO_2_ emission reduction due to wood ash amendment was probably CaCO_3_ formed from CO_2_ and CaO. The reductions in CO_2_ caused by wood ash and lime were very close in magnitude to the change in SIC; therefore, we postulate that the mechanism for CO_2_ emission reduction by lime and wood ash is the reaction of CO_2_ produced during the incubation process with CaO to produces CaCO_3_. The possibility of direct microbial/enzymatic involvement in the reaction of CaO and CO_2_ to produce CaCO_3_ is relatively small.

### Wood ash or lime amendment affects the formation of straw-derived new OC/SIC and SOC content

In this study, lime amendment caused a significant increase in SOC, but wood ash did not; Moreover, while lime greatly promoted the formation of straw-derived new OC (46%), it only increased net SOC by 2.4% compared to that of soil with no amendment (Tables 3 and 4). This finding indicates that the simultaneous addition of straw and lime had a positive priming effect on native SOC (data not shown), Therefore, while adding lime (CaO 5 g kg^-1^ soil, CaCO_3_ content of the tested calcareous soil was 6.7%) can promote the conversion of straw C to new OC, it may also cause a loss of native OC (priming effect). Of course, the potential microbial mechanism underlying the priming effect of straw and lime amendment on native SOC merits exploration in future research.

The maize crop was planted in the tested soil over 10 years, so the soil was regarded as C_4_ soil. The δ^13^C of SOC value of the tested soil (from which SIC was removed) showed a distinct decrease after the addition of straw (C_3_ crop residue) compared to that of soil with no straw amendment (Table 4). Wood ash amendment had no effect on δ^13^C of SOC, but lime significantly reduced δ^13^C of SOC in comparison with that of soil with no lime amendment. It is possible that lime increased the soil pH, which increased the negative charge on molecules of organic matter and eventually increased organic matter solubility [43]. In different studies, liming has been reported to decrease SOC content [44], increase SOC content [45], and leave SOC content unchanged [46]. In this study, the simultaneous addition of straw with lime significantly enhanced net SOC and new OC formation in comparison with soil with no amendment (Tables 3 and 4). The rate and direction of changes in SOC following lime amendment depend on the balance between SOC gains and losses. In our study, it appears that increased C input as a result of lime addition promoted straw degradation, which greatly increased the DOC concentration in the soil treated with straw and lime; this effect may be one explanation for the simultaneous increase in SOC and reduction in C in the remaining straw (Table 5). Moreover, Ca^2+^-mediated formation of soil particle aggregates creates a favorable environment for C accumulation [47]. Lime-induced changes in pH initially increase SOC solubility [30] and may subsequently prevent microbial decomposition by stabilizing SOC via Ca^2+^ bridging.

In this study, with no amendment with exogenous substances no obvious changes in net SIC content occurred before or after the incubation. As mentioned, above, the addition of lime or wood ash increased the net SIC content, which indicated that CaO reacted with CO_2_ to form CaCO_3_ (Table 6), This finding is in agreement with those reported by Zhao et al. [23] and Lee et al. [48].

### Wood ash and lime have significant effects on soil DOC and pH

The high effective CaO content of lime is likely responsible for the greater magnitude of the increase in soil pH produced by lime in comparison with that produced by wood ash. The high soil pH following lime amendment increased the solubility of organic matter in comparison with that of organic matter in soil with no amendment, which resulted in higher DOC content in the lime-amended soil.

## Conclusions

This study demonstrated that simultaneously adding wood ash or lime with returned crop straw delayed the CO_2_ emission peak and reduced the amount of total CO_2_ emitted into the atmosphere. The reduction in cumulative CO_2_ emission was much less with adding wood ash than with lime. The extent of CO_2_ emission reduction caused by wood ash or lime depends on the actual CaO content in each substance. Accordingly, the addition of wood ash or lime enhanced SIC content by 1.4% and 11.8%, respectively. As expected, straw could enhance straw-derived OC, and the lime addition also increased straw-derived OC by 34.5% compared to no addition. In addition, straw addition or lime addition increased the net SOC content by 19% and 2.4%, respectively. Irrespective of straw addition or not, lime addition greatly increased the DOC content compare to no addition at the different period of incubation. During the entire incubation, straw addition decreased soil pH, and lime addition increased soil pH; whereas, wood ash increased soil pH only at the late period of incubation. A possible mechanism for the observed reduction in CO_2_ emission from the tested calcareous soil was CaCO_3_ formation, i.e., the reaction between CaO supplied by wood ash or lime and CO_2_ emitted from the soil. The addition of lime in an appropriate manner can reduce CO_2_ emission into the atmosphere and promote SOC sequestration.
